# Multimorbidity clusters in adults 50 years or older with and without a history of cancer: National Health Interview Survey, 2018

**DOI:** 10.1186/s12877-023-04603-9

**Published:** 2024-01-11

**Authors:** Gabriela Plasencia, Simone C. Gray, Ingrid J. Hall, Judith Lee Smith

**Affiliations:** 1 Epidemiology and Applied Research Branch, Division of Cancer Prevention and Control, Centers for Disease Control and Prevention, Atlanta, GA USA; 2 Department of Family Medicine & Community Health, Duke University Medical Center, Durham, NC USA; 3 National Clinician Scholars Program, Duke University, Durham, NC USA

**Keywords:** Cancer survivors, Multiple chronic conditions, Multimorbidity, Multimorbidity clusters, Cancer health disparities, Factor analysis

## Abstract

**Background:**

Multimorbidity is increasing among adults in the United States. Yet limited research has examined multimorbidity clusters in persons aged 50 years and older with and without a history of cancer. An increased understanding of multimorbidity clusters may improve the cancer survivorship experience for survivors with multimorbidity.

**Methods:**

We identified 7580 adults aged 50 years and older with 2 or more diseases—including 811 adults with a history of primary breast, colorectal, cervical, prostate, or lung cancer—from the 2018 National Health Interview Survey. Exploratory factor analysis identified clusters of multimorbidity among cancer survivors and individuals without a history of cancer (controls). Frequency tables and chi-square tests were performed to determine overall differences in sociodemographic characteristics, health-related characteristics, and multimorbidity between groups.

**Results:**

Cancer survivors reported a higher prevalence of having 4 or more diseases compared to controls (57% and 38%, respectively). Our analysis identified 6 clusters for cancer survivors and 4 clusters for controls. Three clusters (pulmonary, cardiac, and liver) included the same diseases for cancer survivors and controls.

**Conclusions:**

Diseases clustered differently across adults ≥ 50 years of age with and without a history of cancer. Findings from this study may be used to inform clinical care, increase the development and dissemination of multilevel public health interventions, escalate system improvements, and initiate innovative policy reform.

## Background

Multimorbidity is commonly defined as the presence of 2 or more simultaneous diseases in an individual [1]. Multimorbidity is a considerable public health concern due to its impact on health-related quality of life (HRQOL) [2, 3], health care utilization [4], cost of care [4], and mortality rates [5]. The prevalence of multimorbidity in the US adult population has increased from 21.8% in 2001 to 27% in 2018 [6], and it is projected to increase to 50% by 2030 [7]. An aging US population [8] may contribute to this increased multimorbidity prevalence. The percentage of the US population aged 65 and older rose from 12.4% in 2000 to 15.2% in 2016, and it is projected to increase to 21% of the total US population by 2030 [8]. The increase in multimorbidity is also related to the rising number of incident cases of cancer [9] and cancer prevalence [10].

In 2010, to drive changes in care delivery and increase research on multimorbidity, the US Department of Health and Human Services (HHS) developed the *Strategic Framework on Multiple Chronic Conditions* [11]. The framework called for greater understanding of combinations of diseases to inform prevention and management strategies and to improve health and QOL among populations with multimorbidity [11]. However, literature on the prevalence and impact of multimorbidity combinations is limited. There is heterogeneity in (a) terms used (multiple chronic conditions [12], comorbidity [1], and multimorbidity [1]), (b) measurement indices [13], and (c) methods of analysis [14], making comparisons across studies difficult. Nevertheless, studies [15, 16] have shown that diseases co-occur in individuals at rates higher than what would be expected by mere chance. Therefore, it has been suggested that studies examine disease clusters in patients with multimorbidity [17, 18].

The characterization of multimorbidity clusters may provide greater insights about: projected patient outcomes; ways to reduce disease progression; medication or behavioral intervention protocols to enhance patient health and well-being; or community or system changes to reduce risks for poor outcomes, suboptimal disease management, or additional disease diagnoses. Further, since many studies limit their examination to individual diseases or report on the numerical count of diseases, exploring multimorbidity clusters at a population level may provide added information about the health status of the US population. As a result, cluster analysis has emerged as a useful method of understanding the patterns and distribution of multimorbidity [17].

Current US research on multimorbidity clusters has focused on multimorbidity in subpopulations, such as American Indian [19], African American men [20], homeless veterans, and adults aged 65 and older [21, 22]. Kenzik et al. used population-based survey data to assess multimorbidity clusters in cancer survivors aged 65 and older [23]. This study found that multimorbidity clusters were associated with worse functional impairment than multiple unclustered diseases [23]. However, this study did not include a control group of adults without cancer, adults aged 50–64 years, or adults older than 80 years of age. Our study aimed to fill gaps in the current literature by assessing multimorbidity clusters in adults 50 years of age and above, with and without a history of cancer.

## Methods

### Data source

The National Health Interview Survey (NHIS) (https://www.cdc.gov/nchs/nhis/) is a cross-sectional household survey of the US civilian, noninstitutionalized population conducted annually by the National Center for Health Statistics (NCHS) [24]. NHIS collected data on demographics, health-related characteristics, and multimorbidity. In 2018, the final response rate for the sample adult component was 53.1% [24]. More detail about how the sample adult component was selected can be found in the NCHS 2018 Survey Description [24].

### Measures

#### Participants and cancer characteristics

Males and females aged ≥ 50 years without a history of cancer were the control group, hereafter called controls. Males and females aged ≥ 50 years with a history of breast, colorectal, cervical, prostate, or lung cancer were included in the cancer survivor group. These cancer types were selected because they are the most commonly diagnosed in the United States (https://gis.cdc.gov/Cancer/USCS/?CDC_AA_refVal=https%3A%2F%2Fwww.cdc.gov%2Fcancer%2Fdataviz%2Findex.htm#/AtAGlance/), with associated routine, population-level preventive screenings for average risk individuals unanimously recommended by professional and guidance organizations and widely covered by insurance [10]. These cancer types are particularly important for our research question given that screenings for these cancers begin around ages 40–50 for average risk individuals (except cervical cancer screening). Therefore, there is a greater likelihood of initial diagnosis of these cancers between the ages of 50–65, which may impact the health trajectory and clustering of multimorbidities in adults with and without cancer across age groups and inform policy and practice recommendations related to these preventable cancers. Cancer survivors were individuals who responded “yes” to the question, “Have you ever been told by a doctor or other health professional that you had cancer or a malignancy of any kind?” and who self-reported the type as breast, colorectal, cervical, prostate, or lung. Cancer survivors diagnosed before the age of 21 or those who reported multiple cancers were excluded due to differences in treatment exposure and survivorship experience that may impact multimorbidity [25, 26]. Information about time since cancer diagnosis (< 2 years, 2–5 years, > 5 years) and age at diagnosis (< 50, 50–64, 65–74, 75–84, 85 +) was also collected.

#### Multimorbidity

We examined 14 diseases in both cancer survivors and controls. Only diseases assessed using the “Have you ever been told” question stem were included in the cluster analysis to minimize variability in multimorbidity regarding recency of diagnosis. Participants self-reported having ever been told by a doctor or other health professional that they had any of the following: hypertension, coronary heart disease, angina pectoris or heart condition/disease, heart attack, stroke, emphysema, asthma, ulcer, diabetes, liver condition, arthritis, high cholesterol, chronic obstructive pulmonary disease (COPD), and hepatitis. A composite multimorbidity count variable was created that summed the number of diseases reported (including cancer) by each participant.

#### Demographic characteristics

We examined several demographic characteristics including age (50–64, 65–74, 75–84, 85 +), sex (male or female), marital status (married, divorced/widowed/separated, never married/unmarried couple), highest education level (< high school, high school graduate/GED, some college, college graduate or higher), and family income (< $35,000, $35,000–$49,999, $50,000–$74,999, $75,000–$99,999, $100,000 +). The sample included persons from non-Hispanic White, non-Hispanic Black/African American, Hispanic, and additional racial and ethnic minority groups. Due to insufficient sample sizes, American Indian/Alaska Native (AIAN), Asian, or multiple race respondents were combined in the additional racial and ethnic minorities group.

#### Health-related characteristics

Health-related risk behaviors (e.g., smoking, alcohol use, and physical activity) were assessed based on self-reported responses. We used the NCHS recode to classify smoking status. Smoking status was defined as never (smoked < 100 cigarettes in lifetime and no longer currently smokes); current (smoked ≥ 100 cigarettes in lifetime and currently smokes); or former (previously smoked ≥ 100 cigarettes in lifetime but no longer currently smokes) (https://www.cdc.gov/nchs/nhis/tobacco/tobacco_glossary.htm). We used the NCHS alcohol consumption classification as well: abstainer (< 12 drinks in lifetime); former drinker (at least 1 drink in any year but no drinks in the past year); infrequent drinking (1–11 drinks in the past year); light drinking (< 3 drinks per week); moderate drinking (3–14 drinks per week); and heavy drinking (> 14 drinks per week) (https://www.cdc.gov/nchs/nhis/alcohol/alcohol_glossary.htm). For this analysis, we classified alcohol consumption using the terms: abstainer, former drinker, infrequent/light current drinker, and moderate/heavy current drinker— a stratification also used in previous studies [27]. The physical activity variable was defined using the 2008 HHS minimum physical activity recommendation (https://health.gov/sites/default/files/2019-09/paguide.pdf) (the recommendation of record at the time of survey administration)—weekly totals of 150 min of moderate-intensity physical activity or 75 min of vigorous-intensity physical activity. We classified physical activity as: no activity; some activity (< 150 min of moderate-intensity physical activity weekly or < 75 min of vigorous-intensity activity weekly); and met or exceeded (≥ 150 min of moderate-intensity physical activity weekly or ≥ 75 min of vigorous-intensity activity weekly). We also used self-reported status of health (excellent/very good, good, fair/poor) and obesity (body mass index [BMI] ≥ 30 kg/m^2^).

### Analysis

We restricted the analysis to participants with 2 or more diseases (including cancer) and conducted an exploratory factor analysis for cancer survivors and controls. Given that this was intended to be an exploratory analysis of disease clusters and differences in clusters found in adults with cancer compared to controls focused on diseases and cancer status, and not to make nationally representative estimates of these conditions, we treated the NHIS data as a convenience sample; all analyses are unweighted and did not account for the complex survey factors. The cluster analysis used an orthogonal varimax rotation. Factors were extracted with eigenvalues > 1 and retained after rotation if the variance explained was > 5%. Items with a moderate to high loading of at least 0.3 on any factor were retained for the corresponding factor [28]. Items could potentially load on multiple factors.

Frequencies of sociodemographic characteristics, health-related characteristics, and chronic conditions were calculated for individuals within the derived clusters for cancer survivors and controls. Membership in a cluster required having any of the diseases defined by the cluster. All variables were examined using frequency tables, and chi-square tests were performed to determine overall differences in cancer survivors compared to controls. For this analysis, *p* values < 0.05 were considered statistically significant. All analyses were performed using SAS version 9.4 (SAS Institute, Cary, North Carolina).

## Results

### Study sample

Table 1 summarizes sample characteristics by cancer status. Significant differences were observed in age, sex, race and ethnicity, and smoking status between cancer survivors and controls. Controls comprised more 50–64-year-old adults (43.0%) compared to cancer survivors (25.4%). Conversely, cancer survivors had almost twice the percentage of adults aged 85 + (12.3%) compared to controls (6.4%). A greater percentage of cancer survivors were widowed/divorced/separated adults (50.2%) compared to controls (42.8%). More cancer survivors were college graduates or had some college compared to controls (60.2% vs 55.8%).

**Table 1:** Characteristics of Persons with Multimorbidity, NHIS 2018 (*n* = 7580)

	**Cancer Survivors** ***n* (%)**	**Controls *n* (%)**	*p* value
**Age Group**	*n* = 811	*n* = 6769	< .0001
50–64	206 (25.4)	2907 (43.0)	
65–74	295 (36.4)	2277 (33.6)	
75–84	210 (25.9)	1152 (17.0)	
85 +	100 (12.3)	433 (6.40)	
**Sex**			< .0001
Male	304 (37.5)	3058 (45.2)	
Female	507 (62.5)	3711 (54.8)	
**Race/Ethnicity**			0.0002
Non-Hispanic White	613 (75.6)	4854 (71.7)	
Non-Hispanic Black	112 (13.8)	850 (12.6)	
Hispanic	37 (4.56)	607 (8.97)	
People from additional racial and ethnic minorities group^a^	49 (6.04)	458 (6.77)	
**Marital Status**			< .0001
Married	340 (41.9)	3011 (44.6)	
Widowed/Divorced/Separated	407 (50.2)	2889 (42.8)	
Never married/Unmarried couple	64 (7.89)	857 (12.7)	
**Highest Education**			0.03
< High school	96 (11.9)	1023 (15.2)	
High school graduate	225 (27.9)	1953 (29.0)	
Some college	244 (30.3)	1977 (29.4)	
≥ College graduate	241 (29.9)	1781 (26.4)	
**Family Income**			0.439
< $35,000	320 (39.5)	2773 (41.0)	
$35,000–$49,999	103 (12.7)	884 (13.1)	
$50,000–$74,999	155 (19.1)	1149 (17.0)	
$75,000–$99,999	87 (10.7)	658 (9.72)	
$100,000 +	146 (18.0)	1305 (19.3)	
**Self-Rated Health**			0.551
Excellent/Very good	320 (39.5)	2563 (37.9)	
Good	272 (33.6)	2395 (35.4)	
Fair/Poor	218 (26.9)	1808 (26.7)	
**Smoking Status**			< .0001
Never smoker	425 (52.5)	3384 (50.2)	
Current smoker	72 (8.89)	985 (14.6)	
Former smoker	313 (38.6)	2377 (35.2)	
**Alcohol Use**			0.681
Never drinker	165 (20.5)	1273 (19.1)	
Former drinker	189 (23.5)	1619 (24.3)	
Current drinker—infrequent/light	308 (38.4)	2524 (37.9)	
Current drinker—moderate/heavy	141 (17.6)	1247 (18.7)	
**Physical Activity (in past week)**			0.519
No activity	310 (39.3)	2488 (37.8)	
Some activity: < 150 min moderate or < 75 min vigorous	179 (22.7)	1608 (24.4)	
Meets/exceeds activity noted above	300 (38.0)	2482 (37.7)	
**BMI**			< .0001
Obesity (≥ 30 kg/m^2^)	254 (32.0)	2603 (39.9)	
**Cancer Type**			
Prostate	242 (29.8)		
Breast	386 (47.6)		
Colon/rectal	91 (11.2)		
Lung	45 (5.55)		
Cervical	47 (5.79)		
**Time Since Diagnosis**			
Immediate (< 2 years)	107 (13.2)		
Short-term (2–5 years)	192 (23.7)		
Long-term (> 5 years)	512 (63.1)		
**Age at Diagnosis**			
< 50	147 (18.1)		
50–64	358 (44.1)		
65–74	215 (26.5)		
75–84	76 (9.37)		
85 +	15 (1.85)		

^a^People from additional racial and ethnic minorities group includes American Indian persons, Alaska Native persons, Asian persons, persons of multiple races, and persons of other races not releasable

Fewer cancer survivors were current smokers (8.9%) and obese (32.0%) compared to controls (14.6% and 39.9%, respectively). There were no significant differences in distribution of self-rated health status, family income, alcohol use, or physical activity between cancer survivors and controls. The majority of cancer survivors were diagnosed more than 5 years before (63.1%). The survivor sample comprised primarily former breast cancer (47.6%) patients, and most survivors had been diagnosed at ages 50–64 (44.1%).

### Prevalence of multimorbidity

**Fig. 1:**
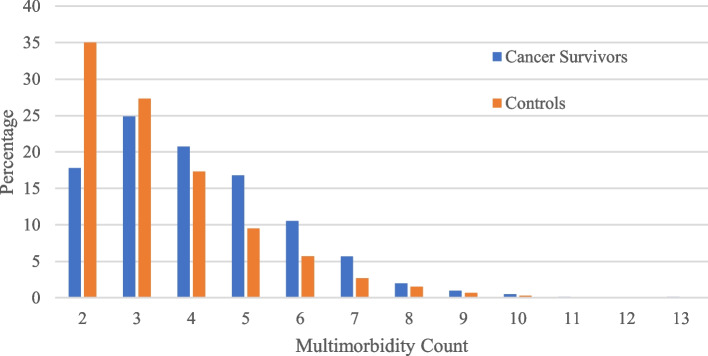
Percentages of multimorbidity count by cancer status

Table 2 summarizes disease prevalence in the sample stratified by cancer status. Controls reported a significantly higher prevalence of hypertension, heart attack, diabetes, arthritis, and high cholesterol compared to cancer survivors, whereas cancer survivors reported higher prevalence of emphysema. There was a significant difference in the distribution of the number of multimorbidities between cancer survivors and controls (Table 2). Cancer survivors had higher prevalence of reporting 4 or more diseases compared to controls (Fig. 1), whereas Table 2 reveals that controls more frequently reported multimorbidity counts of 2–3.

**Table 2:** Prevalence of multimorbidity in persons with at least 2 diseases, NHIS 2018 (*n* = 7580)

	**Cancer Survivors *n* (%)**	**Controls** ***n* (%)**	*p* value
**Individual Diseases**	*n* = 811	*n* = 6769	
Hypertension	569 (70.3)	5097 (75.3)	0.002
Coronary heart disease	114 (14.1)	1011 (15.0)	0.497
Heart attack (myocardial infarction)	58 (7.16)	698 (10.3)	0.004
Angina/other heart condition	185 (22.8)	1557 (23.1)	0.886
Stroke	75 (9.27)	641 (9.48)	0.849
Emphysema	53 (6.56)	324 (4.79)	0.029
Asthma	144 (17.8)	1228 (18.1)	0.828
Ulcer	103 (12.7)	866 (12.8)	0.938
Diabetes	167 (20.6)	1875 (27.7)	< .0001
Liver disease	19 (2.38)	218 (3.26)	0.181
Arthritis	440 (54.4)	4059 (60.0)	0.002
High cholesterol	472 (58.5)	4536 (67.2)	< .0001
COPD	98 (12.1)	751 (11.1)	0.392
Hepatitis	34 (4.24)	365 (5.47)	0.143
**Multimorbidity Count**			
2–3	346 (42.7)	4220 (62.3)	< .0001
4–5	304 (37.5)	1811 (26.8)	
6 +	161 (19.8)	738 (10.9)

### Characteristics of multimorbidity clusters

The cluster analysis yielded 6 clusters for cancer survivors and 4 clusters for controls. Only clusters that matched across both groups were named. These included the pulmonary cluster, with 3 conditions: COPD, emphysema, and asthma; the cardiac cluster, with 3 conditions: coronary heart disease, heart attack, and angina/other heart condition; and the liver cluster, with 2 conditions: hepatitis and liver disease. Additionally, there were 3 unmatched clusters for cancer survivors and 1 unmatched cluster for the controls.

Clusters occurring in both cancer survivors and controls were compared in Table 3. In both the pulmonary cluster and the cardiac cluster, there were significant differences between cancer survivors and controls in age group, sex, marital status, smoking status, and multimorbidity count. Significant differences in race/ethnicity were observed in the cardiac cluster only. In the liver cluster, there were significant differences in age group and multimorbidity count between cancer survivors and controls.

**Table 3:** Characteristics of cluster membership for individuals with 2 or more diseases

	**Pulmonary Cluster**			**Cardiac Cluster**			**Liver Cluster**		
	**Cancer Survivors *n* (%)**	**Controls** ***n* (%)**	*p* value	**Cancer Survivors *n* (%)**	**Controls** ***n* (%)**	*p* value	**Cancer Survivors *n* (%)**	**Controls** ***n* (%)**	*p* value
**Age Group**	*n* = 213	*n* = 1788	0.001	*n* = 247	*n* = 2193	< .001	*n* = 47	*n* = 493	< .001
50–64	70 (32.9)	854 (47.8)		35 (14.2)	753 (34.3)		12 (25.5)	245 (49.7)	
65–74	81 (38.0)	562 (31.4)		85 (34.4)	746 (34.0)		17 (36.2)	183 (37.1)	
75–84	46 (21.6)	271 (15.2)		83 (33.6)	478 (21.8)		14 (29.8)	51 (10.3)	
85 +	16 (7.51)	101 (5.65)		44 (17.8)	216 (9.85)		4 (8.51)	14 (2.84)	
**Sex**			0.018			0.009			0.805
Male	70 (32.9)	738 (41.3)		107 (43.3)	1143 (52.1)		24 (51.1)	261 (52.9)	
Female	143 (67.1)	1050 (58.7)		140 (56.7)	1050 (47.9)		23 (48.9)	232 (47.1)	
**Race/Ethnicity**			0.123			0.004			0.326
Non-Hispanic White	162 (76.1)	1295 (72.4)		184 (74.5)	1641 (74.8)		35 (74.5)	339 (68.8)	
Non-Hispanic Black	28 (13.2)	229 (12.8)		39 (15.8)	239 (10.9)		6 (12.8)	57 (11.6)	
Hispanic	8 (3.76)	151 (8.45)		6 (2.43)	169 (7.71)		5 (10.6)	52 (10.5)	
People from additional racial and ethnic minorities group	15 (7.04)	113 (6.32)		18 (7.29)	144 (6.57)		1 (2.13)	45 (9.13)	
**Marital Status**			0.009			0.003			0.187
Married	89 (41.8)	695 (39.0)		90 (36.4)	943 (43.1)		21 (44.7)	200 (40.7)	
Widowed/ Divorced/Separated	109 (51.2)	825 (46.3)		141 (57.1)	1009 (46.1)		23 (48.9)	210 (42.8)	
Never married/ Unmarried couple	15 (7.04)	262 (14.7)		16 (6.48)	235 (10.8)		3 (6.38)	81 (16.5)	
**Highest Education**			0.626			0.284			0.128
< High school	33 (15.6)	302 (17.0)		30 (12.2)	356 (16.3)		2 (4.26)	78 (15.9)	
High school graduate	56 (26.4)	505 (28.4)		67 (27.2)	626 (28.7)		11 (23.4)	127 (25.9)	
Some college	77 (36.3)	566 (31.9)		81 (32.9)	645 (29.6)		20 (42.6)	157 (32.0)	
≥ College graduate	46 (21.7)	403 (22.7)		68 (27.6)	554 (25.4)		14 (29.8)	128 (26.1)	
**Family Income**			0.612			0.192			0.355
< $35,000	106 (49.8)	830 (46.4)		100 (40.5)	979 (44.6)		19 (40.4)	244 (49.5)	
$35,000–$49,999	21 (9.86)	229 (12.8)		38 (15.4)	275 (12.5)		8 (17.0)	54 (11.0)	
$50,000–$74,999	29 (13.6)	277 (15.5)		51 (20.7)	361 (16.5)		5 (10.6)	74 (15.0)	
$75,000–$99,999	18 (8.45)	159 (8.89)		24 (9.72)	203 (9.26)		6 (12.8)	36 (7.30)	
$100,000 +	39 (18.3)	293 (16.4)		34 (13.8)	375 (17.1)		9 (19.2)	85 (17.2)	
**Self-Rated Health**			0.785			0.397			0.238
Excellent/Very good	59 (27.7)	505 (28.2)		67 (27.2)	651 (29.7)		15 (32.6)	162 (32.9)	
Good	78 (36.6)	613 (34.3)		95 (38.6)	752 (34.3)		19 (41.3)	150 (30.5)	
Fair/Poor	76 (35.7)	670 (37.5)		84 (34.2)	789 (63.0)		12 (26.1)	180 (36.6)	
**Smoking Status**			0.008			0.003			0.222
Never smoker	88 (41.3)	707 (39.7)		115 (46.6)	992 (45.4)		18 (38.3)	193 (39.2)	
Current smoker	28 (13.2)	390 (21.9)		17 (6.88)	317 (14.5)		5 (10.6)	98 (19.9)	
Former smoker	97 (45.5)	684 (38.4)		115 (46.6)	874 (40.0)		24 (51.1)	201 (40.9)	
**Alcohol Use**			0.088			0.475			0.484
Never drinker	43 (20.4)	272 (15.5)		55 (22.5)	417 (19.4)		7 (14.9)	79 (16.3)	
Former drinker	68 (32.2)	497 (28.4)		72 (29.5)	622 (28.9)		11 (23.4)	136 (28.0)	
Current drinker- infrequent/light	69 (32.7)	655 (37.4)		75 (30.7)	759 (35.2)		22 (46.8)	173 (35.6)	
Current drinker- moderate/heavy	31 (14.7)	328 (18.7)		42 (17.2)	355 (16.5)		7 (14.9)	98 (20.2)	
**Physical Activity**			0.294			0.952			0.685
No activity	100 (48.3)	755 (43.4)		107 (44.0)	945 (44.2)		19 (41.3)	199 (41.9)	
Some activity	40 (19.3)	409 (23.5)		57 (23.5)	483 (22.6)		8 (17.4)	105 (22.1)	
Meets/exceeds	67 (32.4)	575 (33.1)		79 (32.5)	708 (33.2)		19 (41.3)	171 (36.0)	
**Obesity**			0.596			0.062			0.866
BMI (≥ 30 kg/m^2^)	82 (39.0)	704 (40.9)		82 (34.0)	861 (40.2)		17 (36.2)	180 (37.4)	
**Multimorbidity Count**			< .0001			< .0001			0.0001
2–3	43 (20.2)	698 (39.0)		24 (9.72)	648 (29.6)		4 (8.51)	195 (39.6)	
4–5	83 (39.0)	637 (35.6)		107 (43.3)	911 (41.5)		22 (46.8)	169 (34.3)	
6 +	87 (40.8)	453 (25.3)		116 (47.0)	634 (28.9)		21 (44.7)	129 (26.2)	

Table 4 displays characteristics of clusters that did not match across cancer survivor and control groups. There were 3 unmatched clusters for cancer survivors. Unmatched cluster 1 for cancer survivors contained 3 conditions: hypertension, high cholesterol, and diabetes. Unmatched cluster 2 for cancer survivors contained 2 conditions: stroke and arthritis. And unmatched cluster 3 for cancer survivors contained 2 conditions: high cholesterol and ulcer. There was 1 unmatched cluster for controls, which contained 6 conditions: hypertension, high cholesterol, arthritis, asthma, diabetes, and ulcer. Further analyses could not be conducted on these groups due to a lack of comparison group. Demographic composition of each unmatched cluster was similar to demographics for cancer survivor and control groups, as seen in Table 1.

**Table 4:** Characteristics of cluster membership for individuals with 2 or more diseases

	**Cancer Unmatched Cluster 1 *n* (%)**	**Cancer Unmatched Cluster 2 *n* (%)**	**Cancer Unmatched Cluster 3** ***n* (%)**	**Control Unmatched Cluster** ***n* (%)**
	Hypertension, High Cholesterol, Diabetes	Stroke, Arthritis	High Cholesterol, Ulcer	Hypertension, High Cholesterol, Arthritis, Asthma, Diabetes, Ulcer
**Age Group**	*n* = 706	*n* = 462	*n* = 510	*n* = 6706
50–64	164 (23.2)	100 (21.6)	120 (23.5)	2887 (43.1)
65–74	269 (38.1)	165 (35.7)	193 (37.8)	2254 (33.6)
75–84	190 (26.9)	126 (27.3)	135 (26.5)	1139 (17.0)
85 +	83 (11.8)	71 (15.4)	62 (12.2)	426 (6.35)
**Sex**				
Male	274 (38.8)	170 (36.8)	200 (39.2)	3018 (45.0)
Female	432 (61.2)	292 (63.2)	310 (60.8)	3688 (55.0)
**Race/Ethnicity**				
Non-Hispanic White	523 (74.1)	362 (78.4)	385 (75.5)	4804 (71.6)
Non-Hispanic Black	108 (15.3)	49 (10.6)	74 (14.5)	845 (12.6)
Hispanic	35 (4.96)	18 (3.90)	21 (4.12)	452 (6.74)
People from additional racial and ethnic minorities group	40 (5.67)	33 (7.14)	30 (5.88)	605 (9.02)
**Marital Status**				
Married	294 (41.6)	189 (40.9)	211 (41.4)	2990 (44.7)
Widowed/Divorced/ Separated	354 (50.1)	241 (52.2)	257 (50.4)	2856 (42.7)
Never married/Unmarried couple	58 (8.22)	32 (6.93)	42 (8.24)	848 (12.7)
**Highest Education**				
< High school	86 (12.3)	61 (13.3)	64 (12.6)	1013 (15.2)
High school graduate	195 (27.8)	121 (26.4)	137 (27.0)	1931 (28.9)
Some college	211 (30.1)	147 (32.0)	164 (32.4)	1958 (29.4)
≥ College graduate	209 (29.8)	130 (28.3)	142 (28.0)	1770 (26.5)
**Family Income**				
< $35,000	277 (39.2)	195 (42.2)	194 (38.0)	2742 (40.9)
$35,000–$49,999	90 (12.8)	51 (11.0)	74 (14.5)	873 (13.0)
$50,000–$74,999	146 (20.7)	97 (21.0)	111 (21.8)	1143 (17.0)
$75,000–$99,999	75 (10.6)	48 (10.4)	50 (9.80)	654 (9.75)
$100,000 +	118 (16.7)	71 (15.4)	81 (15.9)	1294 (19.3)
**Self-Rated Health**				
Excellent/Very good	277 (39.3)	152 (32.9)	195 (38.3)	2538 (37.9)
Good	238 (33.8)	168 (36.4)	168 (33.0)	2379 (35.5)
Fair/Poor	190 (27.0)	142 (30.7)	146 (28.7)	1786 (26.6)
**Smoking Status**				
Never smoker	373 (52.9)	237 (51.4)	256 (50.3)	3365 (50.4)
Current smoker	60 (8.51)	48 (10.4)	51 (10.0)	969 (14.5)
Former smoker	272 (38.6)	176 (38.2)	202 (39.7)	2349 (35.1)
**Alcohol Use**				
Never drinker	148 (21.2)	96 (21.0)	97 (19.2)	1262 (19.1)
Former drinker	169 (24.2)	109 (23.9)	128 (25.4)	1599 (24.2)
Current drinker—infrequent/light	266 (38.0)	180 (39.4)	190 (37.6)	2508 (38.0)
Current drinker—moderate/heavy	116 (16.6)	72 (15.7)	90 (17.8)	1234 (18.7)
**Physical Activity**				
No activity	278 (40.3)	198 (44.1)	196 (39.5)	2458 (37.7)
Some activity	158 (22.9)	105 (23.4)	112 (22.6)	1594 (24.5)
Meets/exceeds	254 (36.8)	146 (32.5)	188 (37.9)	2464 (37.8)
**Obesity**				
BMI (≥ 30 kg/m^2^)	233 (33.8)	160 (35.1)	167 (33.4)	2597 (40.1)
**Multimorbidity Count**				
2–3	264 (37.4)	123 (26.6)	140 (27.5)	4162 (62.1)
4–5	283 (40.1)	200 (43.3)	230 (45.1)	1807 (26.9)
6 +	159 (22.5)	139 (30.1)	140 (27.4)	737 (11.0)
**Cancer Type**				
Prostate	222 (31.4)	138 (29.9)	161 (31.6)	
Breast	337 (47.7)	215 (46.5)	234 (45.9)	
Colon/rectal	77 (10.9)	57 (12.3)	55 (10.8)	
Lung	37 (5.24)	21 (4.55)	33 (6.47)	
Cervical	33 (4.67)	31 (6.71)	27 (5.29)	
**Time Since Diagnosis**				
Immediate (< 2 years)	91 (12.9)	58 (12.5)	69 (13.5)	
Short-term (2–5 years)	173 (24.5)	96 (20.8)	122 (23.9)	
Long-term (> 5 years)	442 (62.6)	308 (66.7)	319 (62.6)	
**Age at Diagnosis**				
< 50	114 (16.1)	86 (18.6)	86 (16.9)	
50–64	321 (45.5)	185 (40.0)	237 (46.5)	
65–74	189 (26.8)	136 (29.4)	138 (27.1)	
75–84	69 (9.77)	43 (9.31)	38 (7.45)	
85 +	13 (1.84)	12 (2.60)	11 (2.16)	

Figure 1 demonstrates the percentage of adults with 2–3 diseases is higher for controls compared to cancer survivors. However, the percentage of adults with 4–10 diseases is higher for cancer survivors compared to controls. Table 5 demonstrates that a higher proportion of cancer survivors age 85 + had 2–3, 4–5, or 6 + diseases (10.7%, 11.8%, and 16.8%, respectively), compared to adults 85 + in the control group (5.57%, 7.56%, and 8.27%, respectively). Notably, in the age 85 + groups, there were twice as many cancer survivors with 6 + diseases (16.8%) compared to controls (8.27%).

**Table 5:** Number of diseases by age groups and cancer status

	Number of Diseases *n* (%)			
	2–3	4–5	6 +	*p* value
Cancer Survivors				0.0001
50–64	117 (33.8)	62 (20.4)	27 (16.8)	
65–74	117 (33.8)	113 (37.2)	65 (40.4)	
75–84	75 (21.7)	93 (30.6)	42 (26.1)	
85 +	37 (10.7)	36 (11.8)	27 (16.8)	
Controls				< .0001
50–64	2006 (47.5)	662 (36.5)	239 (32.4)	
65–74	1340 (31.8)	651 (36.0)	286 (38.7)	
75–84	639 (15.1)	361 (19.9)	152 (20.6)	
85 +	235 (5.57)	137 (7.56)	61 (8.27)	

## Discussion

This study assessed multimorbidity clusters in adults 50 years of age and older with and without a history of cancer. Our study demonstrates that cancer survivors bear a greater burden of co-occurring conditions as the average multimorbidity count is higher in cancer survivors compared to controls. Multimorbidity was defined as having at least 2 diseases, including cancer, because the co-occurrence of at least 1 disease in addition to cancer can impact health outcomes [3]. However, the difference in multimorbidity counts between cancer survivors and controls is not likely explained solely by the inclusion of cancer as a disease, because the discrepancy between multimorbidity counts most frequently reported by each group differed by more than 1 disease. Specifically, controls reported multimorbidity counts of 2–3 significantly more often than cancer survivors, whereas cancer survivors more often reported multimorbidity counts of 4–5 and 6 + . Higher multimorbidity count is associated with increased care utilization and lower HRQOL [29], functional limitations and geriatric syndromes [30], and risk of care dependence [31].

The clusters identified in our study varied by cancer status. Cardiac, pulmonary, and liver clusters emerged across both cancer survivors and controls, but other clusters were observed only among survivors or among controls. The most reported multimorbidity clusters in the literature, particularly among adults 50 and older, include cardiac, musculoskeletal/arthritis, and mental health clusters, with pulmonary and gastrointestinal disorders often included in the mental health cluster [16]. Other studies identified a cardiopulmonary cluster [20, 32], or a pulmonary cluster with other conditions such as osteoporosis and depression [33]. However, findings are dependent on how multimorbidity is defined [2] and the conditions included in the analysis [34]. For example, NHIS 2018 includes arthritis, rheumatoid arthritis, gout, lupus, and fibromyalgia in its arthritis question. However, the Surveillance, Epidemiology, and End Results cancer registry and Medicare Health Outcomes Survey (SEER-MHOS) asks if the patient has ever had arthritis of the hip/knee or hand/wrist [35]. Additionally, in this study, we determined frequency in each cluster by requiring that the individual have at least 1 condition from that cluster, unlike Kenzik et al. [23], which required that people have the majority of conditions in each cluster. Restricting cluster inclusion to those with the majority of conditions in a cluster may bias toward individuals with higher multimorbidity counts and people with more severe disease in that cluster (cardiovascular, pulmonary, metabolic, etc.)—which would be more restrictive and less representative of the general population. Since few studies have analyzed multimorbidity clustering across adults with and without cancer, diseases that cluster differently across both groups may be an important area of future exploration.

Interestingly, in all matched clusters comparing cancer survivors and controls (Table 3), controls consistently feature a higher proportion of individuals in the 50–64 age group. There are several possible explanations for this overrepresentation in multimorbidity clusters at a relatively younger age. Prior literature has found that middle-aged adults, typically considered those between the ages of 40 and 65, experience a significant increase in multimorbidity with age [36] until about age 75, where the number of multimorbid conditions will plateau [37]. However, in the aforementioned systematic review, the majority of studies only included adults up to age 80 years old[37]. Furthermore, many of these studies have been conducted in non-U.S. populations, with significant differences in social risks and needs impacting their populations across the lifespan. Additionally, the lack of data collected in national surveys, such as NHIS, related to time since diagnosis, severity, or treatment of self-reported diseases makes it difficult to explain whether these differences are due to treatment or resolution of disease in older populations. Finally, recall bias may play a role in these differences as diagnoses that occurred earlier in life may be underreported and diagnoses made later in life may be overreported, especially when focused on self-report data from older adults in a cross-sectional study.

### Strengths

The strengths of this study include: first, the use of NHIS data, which is drawn from the US population and includes the fee-for-service Medicare population. Second, analyses of multimorbidity clusters in older adults typically restrict samples to adults aged 65 and older. However, the majority of cancer diagnoses in our population-based sample occurred at ages 50–64, so inclusion of this age group in our analysis was imperative. Multimorbidity has a significant impact on HRQOL [38] and health care expenditures [30] in this age group. Additionally, insurance coverage is not guaranteed for adults age 50–64, which may contribute to age-related disparities in access to care. Therefore, this is a critical age group in which to focus both cancer and noncancer related preventive measures, to improve multimorbidity-related outcomes among older age groups. Third, although we were unable to include all racial/ethnic groups, the inclusion of non-Hispanic Black persons and Hispanic persons increases our understanding of differences in multimorbidity clusters, cancer diagnoses, and sociodemographic factors across race and ethnicity. Fourth, prior studies of multimorbidity clusters include cancer as a multimorbidity [22], analyze symptom clusters in relation to a specific cancer type [39], or do not include a noncancer comparison group [23]. However, significant differences between the cancer survivor and noncancer groups in our study demonstrate that cancer diagnoses are associated with higher multimorbidity overall, as well as certain multimorbidity clusters per demographic factors such as age, sex, and race/ethnicity. Finally, our analysis includes multimorbidity clusters rather than simple counts, dyads, or triads, because multimorbidity counts do not demonstrate which specific combinations of diseases are associated with health care utilization [22, 40], disability [41], or complexity [40]. Therefore, although more difficult to analyze and interpret, multimorbidity clusters can inform more focused, economical, and effective prevention strategies.

### Limitations

The results of a multimorbidity cluster analysis depend on how multimorbidity is defined [2] and the conditions included in the analysis [34]. Due to the lack of consistency in definitions, data sources, and methodology, Goodman et al. [42] proposed a list of 20 diseases to include in future multimorbidity studies. However, we could not utilize this proposed list because not all diseases were assessed directly or used the same question stem in the NHIS 2018 survey. Only conditions assessed using the “Have you ever been told” question stems were included in the cluster analysis, to minimize variability in multimorbidity related to recency of diagnosis. As a result, our analysis did not include the following recommended diseases: autism, chronic kidney disease, dementia, depression, HIV/AIDS, osteoporosis, schizophrenia, or substance use disorder [42]. The exclusion of mental health conditions is a significant limitation given their prevalence in the US and the association between mental health disorders, multimorbidity, and increased incidence of common diseases [43], hospital length of stay [44], and risk of care dependence [31]. Moreover, for individual diseases, NHIS does not include questions about disease severity, management, treatment methods, age at diagnosis, or resolution. Diseases in NHIS are also self-reported, and there is evidence of misalignment between self-reported multimorbidity count and multimorbidity count from other data sources, such as insurance claims, electronic health records, and reports from providers [45, 46].

Of note, we use the term multimorbidity as defined by the presence of 2 or more simultaneous diseases, rather than chronic conditions, in an individual. We also discuss individual disease or diseases, instead of the more commonly used terms—chronic conditions or multiple chronic conditions—because our and other common data sources in the literature do not include temporality of disease diagnosis, treatment, or resolution. Therefore, the chronicity of an individual’s disease is unknown. Furthermore, although the time course for many of the commonly discussed diseases is typically over months and years rather than days or weeks, disease severity or certain treatments can produce transient changes in liver function, kidney function, and blood sugar regulation, to name a few [10, 47].

Additionally, temporal relationships between cancer diagnosis and development of other diseases were difficult to assess due to the lack of information regarding age at diagnosis, severity, and resolution of diseases. Cancer-specific temporal information—including onset of chemotherapy or radiation, severity/staging of cancer, remission, recurrence, and metastasis—is important to understanding the association between multimorbidity and cancer diagnosis [3]. Some of these variables (metastasis, current treatment, and recurrence) were included in the Cancer Supplement in previous years but were not included in the 2018 NHIS survey. Our study excluded: survivors of childhood cancer (defined as those diagnosed with cancer before age of 21); adults reporting multiple cancers; or adults diagnosed with a cancer other than those identified for this analysis. Additional studies can examine multimorbidity clusters in these groups, as different patterns may emerge.

Our study aimed to include several demographic characteristics, including the “oldest old” population and BMI. However, in NHIS, all adults over the age of 85 are coded as 85 (https://ftp.cdc.gov/pub/Health_Statistics/NCHS/Dataset_Documentation/NHIS/2018/samadult_layout.pdf). Therefore, age cannot be used as a continuous variable above the age of 85, which is the age group with the most rapidly increasing incidence of cancer [47]. Additionally, NHIS does not sample institutionalized individuals, such as those in nursing home or other long-term care facilities, which disproportionately impacts the “oldest old” population in the United States. Similarly, BMI was not calculated for all participants because the lowest and highest heights and weights are considered extreme categories and are not included in the data set (https://ftp.cdc.gov/pub/Health_Statistics/NCHS/Dataset_Documentation/NHIS/2018/samadult_layout.pdf). This limitation impacted our ability to provide insight on the oldest old (85 +) population, which is often absent in existing literature. Furthermore, it limited our ability to discuss cachectic and underweight older adults, which can often impact their health, especially among those with cancer [48].

Additional variables associated with multimorbidity clusters in adults older than 65 with and without cancer, such as HRQOL, were not included in this study. The Patient-Reported Outcomes Measurement Information System (PROMIS) questionnaire (https://www.healthmeasures.net/explore-measurement-systems/promis), which measures HRQOL, was previously administered in the Cancer Supplement of NHIS but was not included the 2018 survey. These limitations demonstrate that existing data sources were not created for this type of complex research; thus, it may contribute to the scarcity of cluster analysis and multimorbidity research, especially at a population level.

Finally, our study aimed to understand the differences in disease clusters among adults with cancer and controls. As one of the first studies to use NHIS data for a disease cluster analysis not in relation to a primary diagnosis or within a subpopulation of the US, it was important to analyze diseases in relationship to cancer status, not geographic or demographic distribution. Therefore, the analysis was conducted using data without survey weights. Thus, our results are not generalizable, or nationally representative, and do not account for biases incurred in the sampling process, such as non-response and social desirability bias. Additionally, recall period bias may lead to underreporting of diseases diagnosed at younger ages and overreporting of diseases diagnosed at older ages, especially when focused on self-report data from older adults in a cross-sectional study. Future studies can compare self-report data to other sources of data, such as electronic medical record or claims data, to investigate the impact of recall period bias on self-report of diseases in surveys such as NHIS. However, this was out of the scope of our research.

## Conclusions

Our study aimed to fill gaps in the current literature by assessing multimorbidity clusters in adults 50 years of age and above, with and without a history of cancer. We demonstrated that cancer survivors reported a higher prevalence of having 4 or more diseases compared to controls (57% and 38%, respectively). Furthermore, our analysis identified 6 clusters for cancer survivors and 4 clusters for controls. Three clusters (pulmonary, cardiac, and liver) included the same diseases for cancer survivors and controls. These findings are particularly important given that current clinical trials, guidelines, care management strategies, and health policies overwhelmingly focus on single diseases. Yet diseases not viewed as an individual’s primary disease are often undertreated [16], which can lead to worse health outcomes in individuals with multimorbidity, particularly adults older than age 65 [49] and cancer survivors with other diseases [47].

Identifying patients at risk for multimorbidity clusters may prevent the development of further conditions within a cluster, or conditions that overlap with other clusters. Early identification of these at-risk patients may reduce health care utilization [22], reduce polypharmacy and/or drug interactions [50], and improve case management strategies [48]. Furthermore, tertiary prevention of conditions within multimorbidity clusters has been shown to improve HRQOL among cancer survivors [3] and may improve coordinated care for older cancer survivors [51].

Despite the public health implications of multimorbidity, the aforementioned limitations, which are not unique to our design or data source, may explain why there are so few population-based multimorbidity cluster studies evaluating differences in cancer and noncancer groups in the US. Additional cancer and noncancer related temporal, severity, treatment, and demographic data may allow researchers to further examine the impact of multimorbidity in cancer survivors. If researchers can develop a standard list of diseases (similar to that proposed by Goodman et al. [42]), with an identical question stem format included across national surveys, we could potentially improve comparability of results from multimorbidity studies and identification of health disparities. This standard list could include not only the most prevalent diseases, but also diseases from each organ system—similar to the “Review of Systems” typically performed by physicians—to ensure inclusion of all possible factors contributing to clusters, which would be helpful given the exploratory nature of cluster analysis [18] and the variability of clusters identified based on conditions included [16]. By improving multimorbidity definitions and measurement, and engaging in additional research on multimorbidity clusters, we may have an enhanced understanding about the health status of persons aged 50 and older in the US and may inform multilevel public health action to reduce the burden of multimorbidity.

## Data Availability

The data sets used and/or analyzed during the current study are publicly available at https://www.cdc.gov/nchs/nhis/nhis_2018_data_release.htm.

## Abbreviations

HRQOL: Health-related quality of life
HHS: Health & Human Services
NHIS: National Health Interview Survey
NCHS: National Center for Health Statistics
COPD: Chronic obstructive pulmonary disease
AIAN: American Indian/Alaskan Native
BMI: Body mass index
SEER-MHOS: Surveillance, Epidemiology, and End Results-Medicare Health Outcomes Survey
PROMIS: Patient Reported Outcomes Measurement Information System
